# Advantages Conferred by the Amendment Act

**Published:** 1913-09-20

**Authors:** 


### OUR NATIONAL INSURANCE ACT DEPARTMENT.

### LXVII.—Advantages Conferred by the Amendment Act.

In popular estimation the principal alterations affecting National Insurance effected by the Amendment Act are naturally those which refer to increase of benefits or decrease of contributions. Thus, it is no wonder that attention has chiefly been devoted in the public press to the abolition of reductions of benefit to old persons, the extension of the period for entering insurance at full rates of benefit, and the remission of arrears of employers' contributions. These are the direct ways in which the advantages of increased benefits or reduced contributions have been conferred by the Act.

There are other ways of a less direct character, however, by which insured persons will obtain similar advantages. We propose to consider a few of these in some detail, and to show how some of the less known provisions of the Act will operate to the advantage of the insured.

So few insured persons have become deposit contributors that it is now feasible to use the expression "insured person" as synonymous with "member of an approved society," and it is only when specific reference has to be made to deposit contributors that it is necessary to make any distinction.

### The Benefit of Simplicity.

It should, then, be premised that although certain of the alterations effected by the Act appear at first sight to be merely variations in procedure and method of administration, and therefore to affect only those who are interested as officials of approved societies, these alterations have all been designed with a view to simplifying the administration. This means not only increased efficiency, but also in many cases it will tend to diminish the cost of management, and will thus be to the advantage of the insured. Simplification of details makes it easier to pay benefits promptly, and promptitude in the discharge of obligations is one of the prime essentials of a popular scheme of insurance of industrial workers. A reduction in administrative expense means additional benefit to the members. It may be merely that the reduction will enable the expense to be covered by the allowance prescribed by the Insurance Commissioners for that purpose. But even so it will be a direct gain, for if that allowance is exceeded the members will have to make it good by means of a special levy or in other ways. On the other hand, a reduction in expense of management may mean that an actual saving will be made, and if this be so the members will reap the advantage when the time comes for a valuation to be made and surplus to be distributed. Every alteration, therefore, which tends to simplify the administrative machinery is so much to the good for all concerned.

### Cheap Birth and Marriage Certificates.

Not least among the attractions of the scheme whereby the reductions of benefit to persons over fifty years of age are abolished is the increased facility for determining the rate of benefit.

There were, under the original Act, reductions of benefit for persons over fifty years of age at entry into insurance, and these were graded for persons between fifty and sixty, sixty and sixty-five, and sixty-five and seventy. With each gradation there would inevitably be a strong temptation to misstatement, and to prevent malversation it was frequently necessary to call for the production of birth certificates.

This was foreseen when the Act was passed, and provision was made whereby such certificates could be obtained for the purpose at the specially reduced rate of 6d. In spite of this concession it was annoying, both to the insured and to the officers of his society, that the payment of benefit had to be deferred pending the production of evidence of age. The abolition of the reduction of benefit will thus enable payment to be made more promptly, and will in many cases save the insured the cost of a birth certificate and, perhaps, also a considerable amount of trouble in the not unlikely event of there being difficulty in stating the precise place and date of birth.

While mentioning birth certificates it may be pointed out that the Amendment Act provides (Section 35) that certificates of marriage shall be obtainable at the reduced rate of 1s. if it be necessary to produce evidence of marriage for any purpose connected with National Insurance. It is essential at times to call for production of such documentary evidence of marriage, and the concession is therefore of value, though hardly, perhaps, to the same extent as with certificates of birth, for the tenacity with which the average British woman retains her "marriage lines," in spite of removals of residence, is well known.

### Advantages of Half-Yearly Insurance Cards.

An alteration which will materially affect both the efficiency of management and the cost of administration of approved societies is the substitution of half-yearly for quarterly insurance cards. We referred to this very briefly in our issue last week. This alteration is not directly laid down in the Act, but it is the outcome of the revised method of dealing with arrears of contribution. It may, perhaps, be of interest to point out that there was nothing in the principal Act to require the use of quarterly cards, but this arrangement was deemed to be the most suitable by the Insurance Commissioners in the discharge of their powers regulating the collection of contributions.

The Insurance cards are the property of the Insurance Commissioners, and are printed at the expense of the nation apart from the finance of the approved societies. The saving of expense of printing will not, therefore, affect the societies; but the saving of labour in having to handle one card instead of two, and to make one entry in the contribution register instead of every two formerly required, will be enormous. It will, of course, be necessary to count the stamps for twenty-six weeks instead of for thirteen, but the counting is done principally by way of deduction—that is to say, it is easier to count the number of spaces missed and deduct them from the total possible than to add up the actual stamps on the cards. The extra work entailed by having to count a larger number of stamps will not be considerable, as so few stamps on the whole are missed.

The large approved societies have found it necessary to employ clerical staffs numbering several thousands, and even then great difficulty has been experienced in coping with the work. It may be that some reduction in the numbers employed will be feasible, but in any case the unanimous welcome extended to the scheme by the officers of the approved societies clearly shows that they have substantial hope of being better able to carry out their duties under the Act.

### The Lot of the Collector.

Unfortunately, the Amendment Act appears to have been just too late to enable the alteration to be made as from October 13. The first Insurance books covered the records of contributions and benefits for the first five quarters, and were due to be surrendered next month. It would, therefore, have been a suitable opportunity to change the system of cards at the same time as the Insurance books. There was, however, an even stronger reason for desiring the half-yearly cards to commence as from October. It is probably safe to say that more than one half of the whole of the cards issued are collected by agents or representatives of approved societies from the homes of their members, and according to the arrangements which are apparently to be put into force in January next it will be necessary for the cards to be collected towards the end of January and July each year. At the end of July holidays are in full swing, and this adds to the work of the collector, for he may have to call several times for a given card before he can find the member at home, whereas at other times one call would be sufficient. In a similar way the January collection, particularly in rural districts, will add hardship to the lot of the collector.

Neither of these considerations would apply to the collection in April and October, and it was for this reason that we terminated our remarks last week with the query, "Is it too late even now, although the sixth quarter's cards have been printed, to make arrangements for the half-yearly cards to commence in October?"

### Exemption from Stamp Duty.

Another matter which should give satisfaction is the provision which exempts the payment of stamp duty upon documents in connection with National Health Insurance. A full list of the documents exempted is set out in the second schedule to the Act, and it may be mentioned that the list contains (*a*) draft, order, or receipt given by or to an approved society in respect of money payable under the Act; (*b*) letter or power of attorney given by a trustee for the transfer of money invested in his name; (*c*) bond or other security against malversation by officials of approved societies; (*d*) appointment or revocation of agent; and (*e*) agreement between an approved society and an Insurance Committee in regard to medical benefit.

In this matter the new Act places all approved societies on an equality and follows the precedent of the Friendly Societies Act, which gave exemption from stamp duty on documents as one of the chief advantages of the society becoming a registered friendly society.

**NOTE.—Questions and Correspondence on all doubtful points are invited, and should be addressed to the Insurance Editor, The Hospital, 28 Southampton Street, Strand.**

### Answers to Correspondents.

Nurse M. E. H. asks if it is necessary for private nurses working on the co-operative system to contribute under the National Insurance Act.

The Insurance Commissioners have held that all nurses are compulsorily insurable, the only possible exception being when a nurse receives patients at her own home. Generally speaking, the patient is deemed to be the employer, and is responsible for the payment of contributions, and has the right to deduct the nurse's own contribution from the remuneration.

The liability to be insured is, of course, subject to the limit of the rate of remuneration not exceeding £160 a year. Exemption can be claimed by the nurse from paying her own contributions if she is in receipt of at least £26 a year without personal exertion, or if she is ordinarily and mainly dependent upon some other person, or if she is ordinarily and mainly dependent for her livelihood on earnings derived by her from an occupation which is not employment within the meaning of the Act.

